# Rapamycin Prevents cyclophosphamide-induced Over-activation of Primordial Follicle pool through PI3K/Akt/mTOR Signaling Pathway in vivo

**DOI:** 10.1186/s13048-017-0350-3

**Published:** 2017-08-16

**Authors:** Linyan Zhou, Yanqiu Xie, Song Li, Yihua Liang, Qi Qiu, Haiyan Lin, Qingxue Zhang

**Affiliations:** 1 Department of Obstetrics and Gynecology, Sun Yat-Sen Memorial Hospital, Sun Yat-Sen University, Guangzhou, Guangdong 510120 China; 2 Fertility Center, Shenzhen Zhongshan Urology Hospital, Shenzhen, Guangdong China

**Keywords:** Primordial follicle, Rapamycin, Cyclophosphamide, PI3K/Akt/mTOR

## Abstract

**Background:**

Primordial follicular depletion has thought to be a common adverse effect of chemotherapy especially for female of reproductive age. The study aimed to evaluate the protective effect of rapamycin on the primordial follicles and its potential mechanism for patients receiving chemotherapy.

**Methods:**

8-week old BALB/c female mice were randomly assigned into four groups (control; rapamycin; cyclophosphamide; and rapamycin combined with cyclophosphamide). Hematoxylin staining, immunohistochemical, TUNEL, western blotting and ELISA were employed to assess inter-group differences using Student’s t-test and Mann-Whitney test.

**Results:**

Cyclophosphamide depleted the follicular reserve and induced the phosphorylation of the key proteins of PI3K/Akt/mTOR pathway in mice in a dose-dependent manner. Co-treatment with rapamycin significantly reduced primordial follicle loss at all cyclophosphamide dose groups and prevent the follicle growth wave caused by cyclophosphamide treatment (*P < 0.05*). TUNEL staining showed that no apoptosis occured in the primordial follicles in all groups and fewer apoptosis in large growing follicles were observed in ovaries from rapamycin + cyclophosphamide group compared to that received cyclophosphamide alone. Serum anti-Müllerian hormone (AMH) was significantly reduced in cyclophosphamide alone group, in contrast to the normal level in rapamycin + cyclophosphamide group. Compared to p-Akt/Akt and p-mtor/mtor, p-rps6/rps6 was significantly decreased in rapamycin + cyclophosphamide group (*P < 0.05*), indicating that rapamycin attenuated the increased level of phosphorylation of rpS6 after cyclophosphamide treatment.

**Conclusions:**

Rapamycin can prevent the primordial follicle activation induced by cyclophosphamide through PI3K/Akt/mTOR signaling pathway and thus plays a role in preserving the follicle pool. These results suggest that rapamycin may be an effective protection for ovarian function during chemotherapy, which means a new nonsurgical application for protection of ovarian reserve and prevention of POF.

## Background

Loss of fertility is a disastrous consequence of premature ovarian failure (POF) or insufficiency (POI) [1, 2], which is one of the most significant long-term sequelae of cytotoxic drug treatment. Therefore, maintenance of the ovarian reserve and prevention of infertility are always considered as the priority faced by the patients and their physicians during chemotherapy [3–5]. Although various options have been employed in the efforts for female fertility preservation, including ovarian transplantation, cryopreservation of embryo [6], oocyte [7] and ovarian tissue [8], these methods are subject to a limited use in clinical practice due to the age and conditions of the patients [9], in particular, for pre-pubertal children and single women without a male partner [10]. Besides, as delaying treatment is usually impossible, it is hard to achieve a specific timeframe during which oocyte retrieval is able to be conducted. In addition, transplantation of ovarian tissues back into the patients may carry an additional risk of reintroducing cancer cells that may exist in the grafts [11].

Gonadotropin releasing hormone analogue (GnRHa) has been used to preserve ovarian function during chemotherapy, but there were studies indicating a poor effect of GnRHa in relevant use [12–14]. Therefore, agents would be widely used if they could provide a preventive effect against the loss of follicles induced by cytotoxic drugs but would not weaken antineoplastic effect of cytotoxic drugs. Thus, all females, including children, could benefit from them without invasive operation or assisted reproductive technologies.

Among all kinds of chemotherapy drugs, alkylating agents, such as cyclophosphamide, busulphan and carmustine have the most deleterious effect on ovarian. Cyclophosphamide (CY) has long been used in chemotherapeutic regimes for patients with a wide range of malignancies and autoimmune diseases [15, 16]. Unfortunately, women treated with cyclophosphamide have a high risk of permanent amenorrhea and premature menopause. Ovarian pathological examination usually reveals diminishing primordial follicle pool, ovarian blood vasculature damage and ovarian atrophy in these patients. The underlying mechanisms of these changes still remain unclear. Animal studies have shown that chemotherapy can cause a significant loss in both dormant primordial follicles and growing follicles. Some researchers have also proposed the hypothesis that the increasing activation of follicles from the resting pool leads to accelerating of follicular atresia, which eventually results in a premature “burn-out” of the primordial follicle reserve [17].

The mammalian ovarian reserve is reflected by the primordial follicle pool. A certain number of primordial follicles are continuously activated and then developing into growing follicles while the rest still keep in a dormant state. Therefore, the maintenance of quiescent primordial follicles is believed to be able to determine the reproductive lifespan of females [18]. When primordial follicle pool is exhausted, menopause or ovarian senescence occurs, which may subsequently cause infertility. Some pathological conditions would aggravate the loss of primordial follicles, and eventually lead to premature ovarian failure (POF) [19].

A series of works have been employed to explore the molecular mechanisms behind the quiescence and activation of primordial follicles over the recent years. Researchers have found, mostly in genetically modified mouse models, that phosphatidylinositol 3-kinase/Akt/mammalian target of rapamycin (PI3K/Akt/mTOR) signaling pathway is indispensable for the control of activation, survival, atresia and loss of primordial follicles [20]. Pten (phosphatase and tensin homolog deleted on chromosome ten) [21], Tsc1 (tuberous sclerosis complex1) [22] and Tsc2 [23] are negative regulators of this signaling pathway. Severally deletion of these genes from the oocytes could lead to the premature activation of the entire pool of primordial follicles, which subsequently resulted in follicular depletion in early adulthood in the mutant mice. These models found the increased level of the phosphorylation of ribosomal protein S6 (rpS6) [24] which is activated by mammalian target of rapamycin complex 1 (mTORC1) through multiple mechanisms. In addition, the knockout of PDK1 in oocytes results in increased apoptosis of dormant follicles. These findings allow us to better understand the mechanisms underlying the impact of chemotherapeutic agents on the ovary, possibly providing some new solutions to protect the ovary against chemotherapy-induced damage.

As many chemotherapy regimens induce follicular depletion, fertility preservation became a major concern in young cancer patients. A more thorough understanding of the mechanism behind chemotherapy-induced follicle loss is necessary to develop new methods to preserve fertility in these patients. The pool of primordial follicles in the mammalian ovary represents the ovarian reserve, and the female reproductive lifespan depends on the maintenance of the majority of primordial follicles in a quiescent state. Although the study of primordial follicle is not enough, it is helpful for us to understand the mechanism of premature ovarian failure and infertility. The latest research shows that rapamycin, as an inhibitor of mTOR pathway, can inhibit the excessive activation of primordial follicles of PTEN gene knockout rat ovaries and reduce consumption [21], which helps to maintain the ovum and ovarian reserve function. The present study focused on the inhibitory effect of rapamycin on the activation of primordial follicles, but little research about the effect of rapamycin on the primordial follicles in the ovary after chemotherapy. With the continuous exploration of the mechanism of rapamycin in the ovary, it will bring a new prospect in the prevention and treatment of premature ovarian failure caused by chemotherapy.

We put up with the hypothesis that cyclophosphamide leads to excessive activation of primordial follicle through the PI3K/Akt/mTOR signaling pathway and rapamycin prevents cyclophosphamide-induced over-activation of the primordial follicle pool.

## Methods

### Mice

8-week-old BALB/c female mice (*n* = 118) were purchased from the Center of Experimental Animals, Sun Yat-Sen University. They were housed under controlled conditions of temperature (20–26 °C), relative humidity (35–75%) and photoperiod (12 L:12D), with free access to food and tap water.

Experimental protocols were approved by the Institutional Animal Care and Use Committee (IACUC) of Sun Yat-Sen University.

### Experimental protocol

Cyclophosphamide pulveres (Sigma) were dissolved in phosphate buffered saline (PBS) and prepared into different concentrations before use. 50 mg/ml of Rapamycin (Gene Operation Datasheet) supplied in dimethyl sulfoxide (DMSO) under −20 °C was diluted in a buffer containing 0.2% sodium carboxymethyl cellulose (CMC) and 0.25% Tween 80.

After 3 days of quarantine, animals were randomly assigned into different groups. In order to establish the optimal CY-induced POF models and explore the effect of CY on adult mouse ovaries, the mice received 0.1 ml of single CY injection at different doses (75 mg, 100 mg, 150 mg per kg body weight) as well as repeated injection for 4 weeks (75 mg/kg). The mice in control group were treated with equal volume of PBS. For exploring the interaction between rapamycin and CY in adult mouse ovaries, the mice were administered with 0.1 ml of rapamycin (5 mg/kg) once a day from 1 week before CY treatment to1 week after the final CY administration. Their body weight was monitored daily during and after rapamycin treatment. The mice were euthanized 7 days after the last injection of CY or 24 h after the last rapamycin administration. Besides, 0.1 ml CY (150 mg/kg) was given to mice with or without 0.1 ml of rapamycin (5 mg/kg), and they were euthanized 4 h, 12 h or 24 h after drug administration. Ovaries were removed from the mice and placed immediately into 4% paraformaldehyde (PFA) for histological follicle assessment or store in −80 °C for western blotting. Before the mice were euthanized, blood samples were collected from eyeballs and centrifuged to obtain the plasma, which was then cryopreserved under −80 °C for ELISA test.

### Ovarian Histomorphology

The ovaries were fixed in 4% PFA for 24 h, and then dehydrated, paraffin-embedded, and cut into 5 um sections. The sections were rehydrated and stained with hematoxylin and eosin (HE) for morphological observation and differential follicle counts. Blind follicle counting was performed on every fifth sections of the ovaries by two independent investigators. Follicle stages were identified as the following definitions: the primordial follicle is counted when the nucleus is surrounded by a flat layer of pregranulosa cells; primary follicle is an oocyte encircled by a single layer of cuboidal granulosa cells, secondary follicle has at least two layers of cuboidal granulosa cells without antrum and antral follicle has antrum. Both primary follicle and secondary follicle are defined as early growing follicles. Occasionally, follicles may have both of cuboidal and flat squamous granulosa cells, which indicates an intermediate stage between the primordial and primary stages. In this case, stage identification was decided according to the type of predominated granulosa cells.

### Immunohistochemistry and immunofluorescence

After deparaffinization and rehydration, the sections were incubated in citrate buffer (pH 6.0) and heated by microwave for 10 min, and then immersed in 3% H_2_O_2_ for 10 min at room temperature to block endogenous peroxidase activity. Nonspecific binding was blocked with 5% normal goat serum for 1 h under 37 °C. The sections were incubated in a 1:400 dilution of the primary antibody [phosphor-Akt (Thr-308), phosphor-mTOR (Ser-2448) and phosphor-rpS6 (Ser-235/236) (Cell Signaling Technology), Ki67] for 24 h at 4 °C followed by room temperature placement for 30 min. For immunohistochemistry, horseradish peroxidase (HRP) combined secondary antibodies were added for a 30-min reaction in 37 °C, followed by diaminobenzidine (DAB) staining. The sections were counterstained with hematoxylin and then dehydrated and mounted with neutralbalsam. For immunofluorescence, Alexa Fluor 488-conjugated secondary antibodies were added in 37 °C for 1 h, followed by 4,6-diamidino-2-phenyiindole (DAPI) staining for 5 min. The sections were washed three times (5 min for each time) with PBS after each of the above procedure. At the same time, a negative control was treated by replacing primary antibodies with PBS.

### TUNEL assay

In Situ Cell Death Detection Kit-POD (Roche), a commercial kit of TUNEL assay, was employed to evaluate the cell apoptosis of follicles. After dewaxed and rehydrated, the sections were incubated in citrate buffer (pH 6.0) and heated by microwave for 10 min, and then rinsed twice with PBS. 50 ul TUNEL working solution per sample was mixed with label solution and enzyme solution, while negative control used label solution. The sections were incubated in 37 °C for 60 min in the dark, followed by rinsing twice with PBS and analyzed by fluorescence microscopy.

### Blood plasma measurements

AMH ELISA (enzyme-linked immunosorbent assay) kit (Cloud-Clone Corp) was employed to quantify the plasma AMH concentration. The blood samples were put in room temperature and mixed completely before examination. Reference material, quality control serum and blood samples from mice was added to the micropores respectively and then incubated in 37 °C for 1 h. The samples were treated with developer for 15 min before termination of dyeing and the spectrophotometry was performed in 30 min.

### Western blotting

Mouse ovaries were placed on ice about 30 min and then pulverized by ultrasonic wave, and subsequently extracted by RIPA lysis buffer (P0013B, Beyotime Institute of Biotechnology, Shanghai, China) with protease inhibitor PMSF (ST506, Beyotime Institute of Biotechnology). Protein content was determined using a BCA Protein Assay Kit (ComWin Biotech Co., Ltd), and 30μg of the protein from each sample was loaded onto 6–10% SDS-PAGE. The interest proteins were separated by electrophoresis, and transferred to polyvinylidenefluoride (PVDF) membranes. The membranes were then blocked in 5% bovine serum albumin solution (dissolved in tris buffered saline containing 0.1% Tween 20) for 1 h and probed with specific primary antibodies overnight at 4 °C. Primary antibodies against Akt, phosphor-Akt (Ser473), mTOR, phosphor -mTOR (Ser2481), rpS6, phosphor - rpS6 (Ser235/236) and β-actin were all rabbit monoclonal antibodies and purchased from Cell Signaling Technology. Horseradish peroxidase (HRP) -conjugated goat anti-rabbit IgG (7074, CST) was used to determine the proteins and an ECL kit (Pierce, Thermo Scientific) was employed to visualization. ImageJ (National Institutes of Health, Java image processing software) was used to quantify the integrated light intensity of each band,determine the concentration of phosphoproteins as well as the changes induced by treatment, and calculate the ratio of phosphorylated proteins to their nonphosphorylated forms. β-actin expression was measured to verify equal loading.

### Statistics

All experiments were carried out at least three times. Data were expressed as means ± SD and analyzed by Student’s t-test and Mann-Whitney test. *P* < 0.05 was considered statistically significant.

## Results

### CY treatment induces short-term follicle activation and follicle loss

**Fig. 1:**
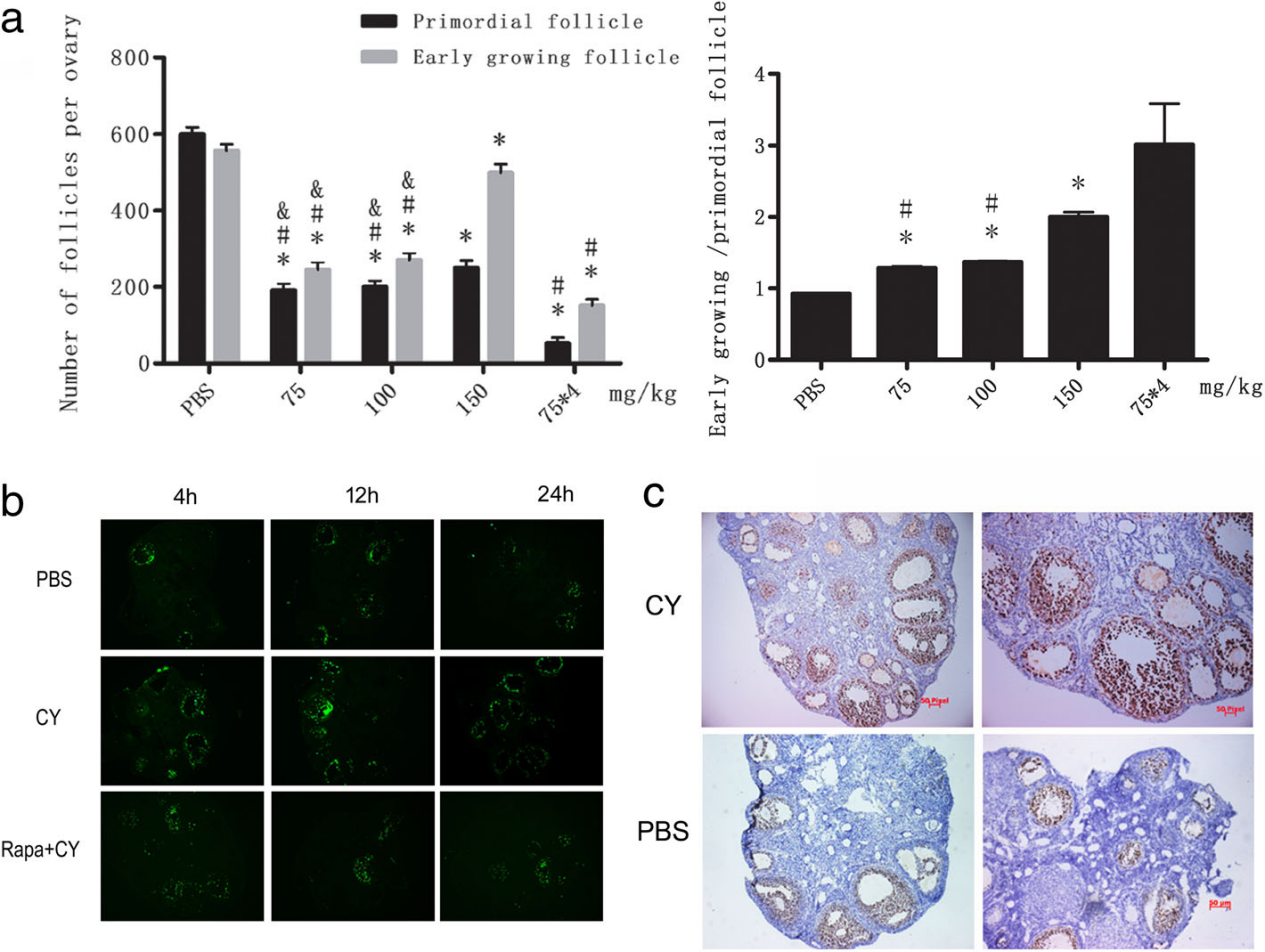
CY treatment induces short-term follicle activation and loss. **a** Changes in the number and the ratio of early growing and primordial follicles in ovaries removed from adult (8-week-old) female BALB/c mice one week after treatment with PBS or varying doses of CY (*n* = 15). (A1) Differential follicle counts of primordial and early growing follicles. Data are presented as means ± SD. (A2) Inter-group comparison on early growing/primordial follicle ratio (*P < 0.05 for the comparison between CY-treated group and PBS control group; #*P < 0.05* for the comparison between 150 mg/kg with other groups; & *P* < 0.05 for the comparison between 75*4 group with other groups). **b** TUNEL staining conducted in CY-treated group (150 mg/kg), PBS group and co-treated group (CY and Rapa) for apoptosis observation using ovaries removed 4, 12, and 24 h after treatment. **c** Ki67 staining conducted in CY-treated group (150 mg/kg) and PBS group for cell proliferation study using ovaries removed from 8-week-old mice 24 h after treatment

In order to establish the optimal CY-induced POF models and explore the effect of CY on adult mouse ovaries, the mice received 0.1 ml of single CY injection at different doses (75 mg, 100 mg, 150 mg per kg body weight) as well as repeated injection for 4 weeks (75 mg/kg). The ovaries were removed 1 week after the final administration. As described above, ovarian follicles were categorized into quiescent primordial follicles and early growing (primary and secondary stage) follicles. The results of follicle quantification 1 week after CY treatment showed that compared to PBS group, the number of dormant follicles and growing follicles were significantly decreased in all CY groups (*P < 0.05*), with the most significant changes observed in the four-week treatment group. By comparison to early growing follicles, dormant follicles were found to suffer a significantly greater loss at all dose groups. The difference of the ratio of early growing follicles to dormant follicles was statistically significant between CY groups and PBS group (*P < 0.05*), and increasing doses of CY was associated with a higher early growing/ dormant follicles ratio (Fig. 1a).

To illustrate CY activated primordial follicle but not destroyed them directly, TUNEL staining was employed to explore whether apoptosis was involved in follicular growth. Ovaries were harvested after 4, 12 and 24 h of the last CY treatment. Preovulatory follicles showed strong positivity of TUNEL sign, especially in the granulosa cells. However, there was no green fluorescence in the primordial follicles at all, which means no apoptosis occurred in dormant oocytes and pregranulosa cells. In case that the number of primary and secondary follicles remained steady or increased, TUNEL positivity in these stages was focused. The number of apoptotic cells were found to increase in a time-dependent manner after CY administration, and the apoptosis in CY groups was significantly elevated after 24 h compared with the PBS group (Fig. 1b).

The function of granulosa cells is important for the survival of follicles, so proliferation immunostaining was conducted in ovaries from 8-week-old mice after CY treatment to detect the follicles that were undergoing growth. The proliferation marker Ki67 was used to dye the ovaries removed 24 h after CY administration and granulosa cell proliferation was observed in early growing follicles. But in control groups, positive result was found in larger growing follicles and occasionally observed in small activated follicles (Fig. 1c).

### PI3K/Akt/mTOR signaling pathway is activated after CY treatment

**Fig. 2:**
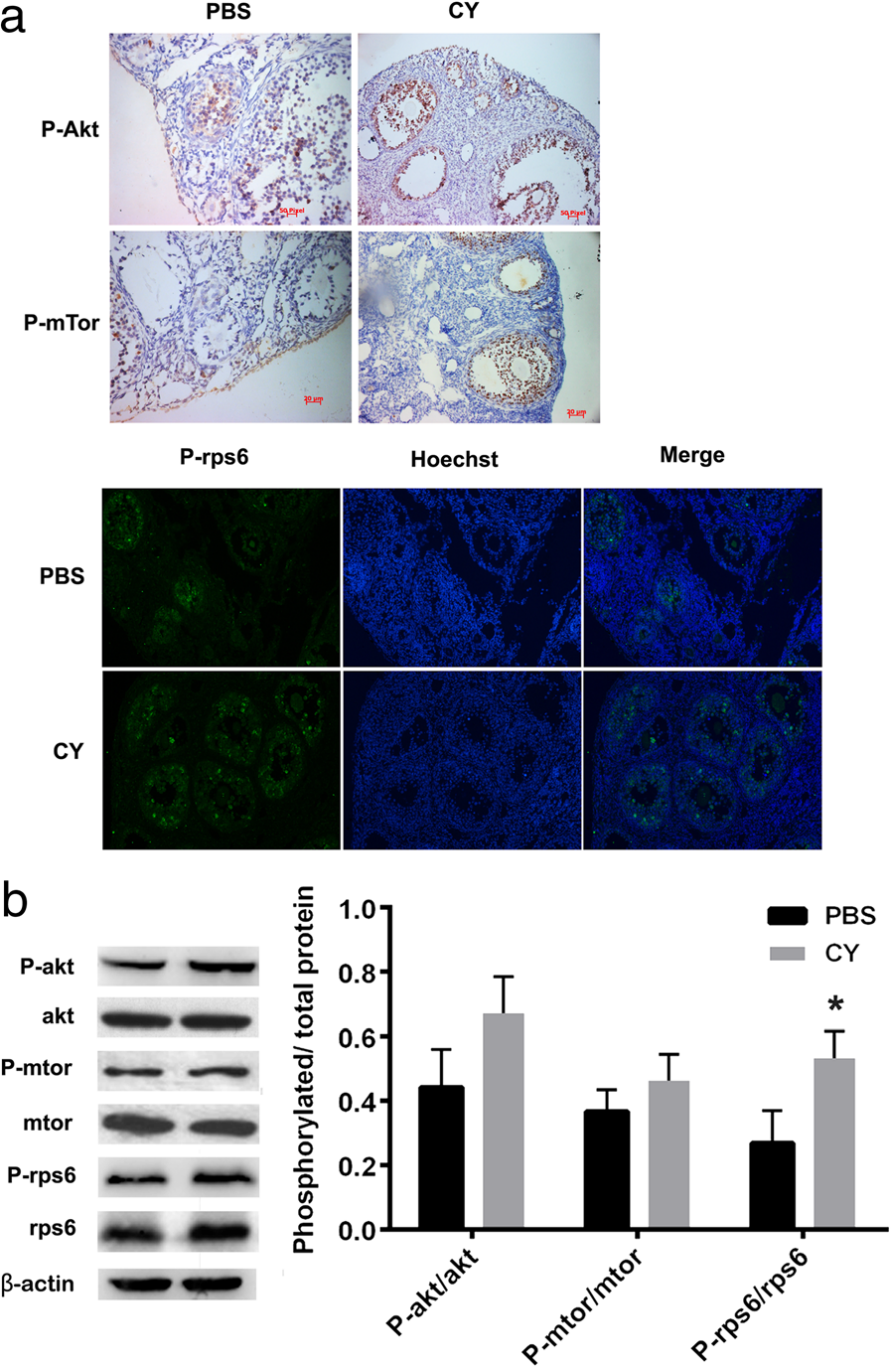
CY treatment activates phosphorylation of key proteins in the PI3K/Akt/mTOR pathway. **a** Immunohistochemistry and immunofluorescence reveal the localization of phospho-Akt, phospho-mTOR and phospho-rpS6 within the oocytes of activated small follicles. **b** Protein analysis demonstrates an significant increase in the phosphorylated rpS6(*P = 0.025)and slightly increased phosphorylation level of Akt and mTOR. β-actin was used as a loading control. The bar graph shows the results from a single experiment out of the three repeated experiments with similar results; Four ovaries were pooled per result, with a total number of animals of 24

Previous studies have shown that the recruitment and depletion of primordial follicle was related to PI3K/Akt/mTOR pathway by increasing phosphorylation of the relevant key proteins [23]. In the present study, the effect of CY on the PI3K/Akt/mTOR signaling pathway was investigated based on the increased proportion of growing follicles. Whole ovaries from 8-week-old mice were removed 24 h after CY or PBS treatment and used for immunohistochemistry and immunofluorescence staining. Brown granules which revealed the localization of the key proteins of the PI3K/Akt/mTOR signaling pathway were seen in the cytoplasm of the oocytes and granulosa cells of activated small follicles in CY group, in contrast to the minimal staining seen in the PBS control sections (Fig. 2a).

Western blotting was conducted to evaluate the changes in phosphorylation of the key activation proteins, including Akt, mTOR, rpS6 and their phosphorylated form. Compared to PBS group, CY treatment group showed an significant increase in the phosphorylated forms of rpS6 (*P* = 0.025), and slightly increased phosphorylation level of Akt and mTOR without statistical difference (Fig. 2b).

### Rapamycin reduces CY-induced follicle loss

**Fig. 3:**
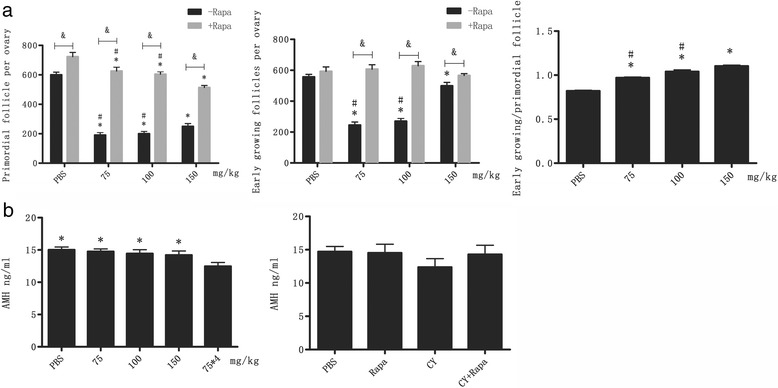
CY-induced follicle loss and decrease in AMH concentration were reversed by Rapa co-treatment. **a** Differential follicle counts on ovaries from 8-week-old female BALB/c mice one week after CY treatment at varying doses with or without Rapa co-administration (total *n* = 12). Data were presented as means ± SEM. *P < 0.05 for the comparison on the same follicle type between CY-treated group and PBS control group; #*P < 0.05* for the comparison on the same follicle type between 150 mg/kg with other groups; *&P < 0.05* for the comparison between −Rapa group and +Rapa group. (A3) Ratio of growing follicles versus dormant follicles in ovaries from CY-treated mice at varying doses (75, 100, and 150 mg/kg) with or without Rapa co-administration. **b** AMH plasma concentration measured after single injection with varying CY dose (75, 100, 150 mg/kg) and 4-week treatment with 75mg/kg, as well as PBS alone, Rapa alone (5 mg/kg on alternative days), or CY (150 mg/kg) with or without Rapa (5 mg/kg) on alternative days starting 1 week before CY treatment. Samples were collected 1 week after the final CY administration (Total n = 28). *P < 0.05 for the comparison on the same follicle type between CY-treated group and PBS control group

The present study showed that rapamycin treatment significantly reduced primordial follicle loss at all CY groups (75 mg/kg, 100 mg/kg and 150 mg/kg) and prevent the follicle growth wave caused by CY treatment, with measurements of the number of primary and secondary follicles similar to those seen in untreated ovaries (Fig. 3a). The numbers of follicles in various stages were not significantly different when the mice were administrated with rapamycin alone. TUNEL staining showed that fewer apoptosis of large growing follicles were observed in ovaries in the rapamycin and CY co-treatment groups compared to CY alone group where extensive apoptosis of granulosa cells was spotted in large growing follicles. No apoptosis was detected in oocytes or pregranulosa cells of primordial follicles in any of the ovaries (Fig. 1b).

Serum anti-Müllerian hormone (AMH) concentration, as an indirect indicator of follicle reserve, was measured in 4-week CY treatment group. AMH was significantly reduced in CY alone group as compared to the normal level (15 ng/ml) in rapamycin and CY co-treatment groups, indicating that rapamycin alone does not change the concentration of serum AMH (Fig. 3b).

### Rapamycin reverses CY-induced over-activation of rpS6 in oocytes

**Fig. 4:**
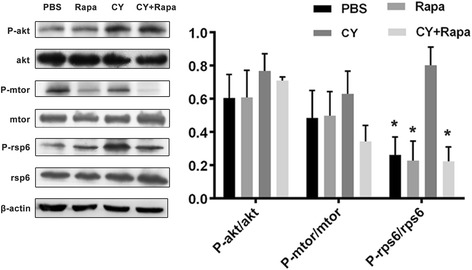
Rapa inhibited CY-induced activation of PI3K/Akt/mTOR pathway proteins in the ovary. Protein analysis on ovaries from 8-week-old mice removed 24 h after CY treatment (150 mg/kg) or PBS treatment with or without co-administration of Rapa. Western blots of phosphorylated and total Akt, phosphorylated and total mTOR, phosphorylated and total rps6, as well as the calculated fold change for each of the proteins. Experiments were repeated three times with similar results (two to four ovaries were pooled per result; total number of animals = 48, with 8 animals in each treatment group and time point) (*P < 0.05 for the comparison on the same follicle type between 75*4 group and other groups)

Western blotting was performed on ovaries removed from 8-week-old mice 24 h after a single dose of CY (150 mg/kg) or the equivalent volume of PBS with or without co-treatment with rapamycin. Phosphorylated and total Akt, mTOR, and rpS6 were analyzed and the fold change of each protein was calculated with the results presented as a bar graph. Compared to p-Akt/Akt and p-mtor/mtor, p-rps6/rps6 was significantly decreased in CY + Rapa group, indicating the level of phosphorylation of rpS6 in oocytes was suppressed by mTOR specific inhibitor rapamycin, which is comparable to the control group almost approach to the normal level from control group (Fig. 4).

## Discussion

### The effect of cyclophosphamide on ovarian reserve

As one of the most commonly used antineoplastic drugs with a potential risk to the gonad, cyclophosphamide imposes a negative effect on gonad in a dose- and age-related manner as demonstrated by the observed higher risk of amenorrhea in aged patients or those on a larger dose of chemotherapeutic regimen. Study of the pharmacological mechanism of cyclophosphamide has revealed that it primarily targets the cell in proliferating phase by intracellular DNA crosslinking, which results in an effect of inhibiting cell growth and promoting apoptosis. Therefore, the major consequence of the impairment to ovaries is considered to be the atresia of follicles in proliferation, whereas the toxicity of cyclophosphamide to quiescent primordial follicles is still unclear. The preliminary results of our study have shown that CY-induced POF do lead to the atresia of the follicles in proliferation phase as well as ovarian interstitial fibrosis. We also found that despite of the increased ratio of early follicles to primordial follicles, apoptosis of the primordial follicles in ovarian cortex was not observed by TUNEL test, which suggested a relative increase in the number of the early follicles since they can only be formed via the recruitment of primordial follicles. These findings have provided indirect evidence supporting that chemotherapy drugs may prematurely induce the primordial follicles into the recruitment stage. The premature recruitment of primordial follicles into the growing follicular pool may be caused by a direct CY-induced apoptosis of granulosa cells of mature antral follicles and a subsequent decrease in the negative regulatory factors for primordial follicle recruitment [16]. Since the growing follicles are more sensitive to chemotherapy agents, the apoptosis process is accelerated by cyclophosphamide, eventually precipitating a rapid decline in ovarian reserve and, as a result, POF. The other possibility is that the direct chemotherapeutics-induced injuries to the DNA of oocytes in primordial follicles may accelerate the repair of defective DNA, and then trigger the premature recruitment of primordial follicles [25].

Oocytes and granulosa cells are different in terms of their susceptibility to chemotherapy drugs, as proliferation and differentiation would not occur in the oocytes even in the follicles at a rapid growth stage, which is contrasted with the continuous proliferation of granulosa cells. In vitro studies have shown that the target of cyclophosphamide leading to ovarian impairment is the granulosa cell whose apoptosis induced by activation of some certain signals in follicles will precipitate POF characterized by the loss of reproductive and endocrine functions of the ovary [26, 27]. So, the first explanation as described above appears to be a more plausible mechanism that the direct CY-induced mature antral follicle apoptosis contributes to the attenuation of the negative regulation factors for primordial follicle recruitment (such as AMH), which, in turn, can cause the premature recruitment of primordial follicles.

8-week old adult BALB/c mice were used in the present study, as the ovarian morphology and follicular development in mouse share a high similarity with those of human. Both of single administration and consecutive 4-week multiple dosing were employed for POF model group to observe the acute effects of cyclophosphamide on ovary as well as to mimic the clinical ovarian changes of patients after repeated dosing of chemotherapeutics. The process of recruitment of primordial follicles to ovulation takes about 9 months, during which several courses of chemotherapy may have been conducted for a patient while it takes only 3 weeks for the mice to go through the same process. Therefore, the models established by consecutive 4-week chemotherapy could be used to observe the effect on the growth and apoptosis of the follicles.

HE staining follicle counts showed that with the increased dose of cyclophosphamide, a constant shrink in the number of antral follicles was observed in contrast to an elevating tendency in the ratio of early ovarian follicles to primordial follicles, which was the most significant in the 75 mg/kg × 4 weeks group. This finding suggests that the damage to ovarian tissues and the decline in the number of follicles, particularly the mature antral follicles, are correlated with the dose of cyclophosphamide. Different degree of fibrosis was also observed in ovarian cortex, which is consistent with the ovarian toxicity from previous reports [2]. Ovarian morphological study for different dose groups revealed that the mice in 150 mg/kg group had the most significant decrease in the number of mature antral follicles and ovarian fibrosis changes as pathologically defined by POF, so 150 mg/kg was selected as the drug concentration for the following mechanism study. It is believed that the models were well-established due to the short modeling time and the ovarian histological changes similar to human POF, and could also be employed in the following studies.

A number of studies have shown that [28, 29] apoptosis of growing cells plays a key role in chemotherapeutics-induced impairment on ovarian structure and function. The TUNEL staining in this experiment also showed that apoptosis of the granulosa cells in antral follicles initiated in CY group within 12 h after drug administration, and significantly larger number of apoptotic follicular granulosa cells were observed compared with the PBS control group after repeated drug administration. However, apoptosis of primordial follicles was not found in any of the groups with different dosing duration or at different doses. In Ki67 immunohistochemical test, high magnification microscopy for the parafin sections showed the most evident DAB staining of Ki67 in granumosa cells of early follicles in CY group, but no DAB stains in the granumosa cells of the follicles at the same stage in PBS group. These findings suggest that cyclophosphamide may directly trigger mature follicle apoptosis, accompanied by an increase of the ratio of early follicles to primordial follicles. The absence of primordial follicle apoptosis and the significant rise in the number of early follicles also indicate that cyclophosphamide may prematurely induce primordial follicles into the growing follicle pool and eventually result in the shrink of ovarian reserve.

Ovarian tissue proteins of the mice in 150 mg/kg CY group were extracted for quantitative determination to explore the correlation between CY-induced effect on ovarian reserve and the signaling pathway closely related to follicular growth. The results demonstrated an increase in phosphorylation of signaling pathway proteins AKT and mTOR but with the absence of statistical difference, as well as a statistically significant elevation in phosphorylation of rpS6, the downstream protein of mTOR (*P = 0.025*), which indicates that cyclophosphamide may cause over-activation of primordial follicles by activating mTOR signaling pathway for follicle growth regulation. However, this conclusion is subject to a certain level of limitations, as the experiment techniques are insufficient to separate the primordial follicles and the early growing follicles in ovarian cortex for protein quantitative determination. Immunohistochemistry and immunofluorescence localization tests were also performed to screen the confounding results caused by mature follicles. Evident staining was observed in the proteins related to this signal pathway in ovarian granulosa cells but not in ovarian interstitial cells, indicating that the follicular granulosa cells may contribute to the enhanced protein phosphorylation.

### The inhibitory effect of rapamycin on chemotherapy-induced ovarian reserve decline

Rapamycin (Rapa), a specific inhibitor of mammalian target protein (mTOR), controls cell proliferation and apoptosis by regulating a variety of biosignals such as saccharides, amino acids and insulin [30]. There are two isomers of mTOR playing different roles in the cells, with mTOR1 mainly responsible for the control of cell growth and mTOR2 for the regulation of cell survival. It has been proved that only mTOR1 can inhibit the activation of downstream signaling molecules and the subsequent cell proliferation by specific binding with rapamycin. Adhikari et al. found that knockout of tuberous sclerosis complex (Tsc) 1 and 2 in oocytes of mice was associated with the elevation in mTOR1 activity and a rapid transform of primordial follicles into growing follicles ultimately leading to premature ovarian follicle depletion and POF [31]. This study suggests that rapamycin may participate in the regulation on the recruitment, proliferation and differentiation of primordial follicles. In 2009, Randy et al. reported that rapamycin may have an anti-aging effect as demonstrated by 9% lifetime extension observed in male mice and 14% in female mice, which was considered as the evidence to support that mTOR signaling pathway may be involved in puberty initiation and lifetime extension [32, 33]. All these findings suggest that rapamycin is closely associated with the recruitment of primordial ovarian follicles, proliferation and apoptosis of follicles, as well as the anti-aging mechanisms.

In vivo study using gene knockout mice has shown that rapamycin as the specific blocker of mTORC1 can inhibit the recruitment of primordial follicles in the ovaries of mice [21]. In the present study, comparison between concomitant rapamycin administration 1 week before and after chemotherapy and cyclophosphamide alone regimen revealed a statistically significant decline in the ratio of early growth follicles to primordial follicles in mouse ovaries, suggesting that rapamycin may inhibit the recruitment of primordial follicles in chemotherapeutics-treated mouse models. According to the references, the anti-apoptosis effect of rapamycin is considered to be achieved by involving the cell division cycle from G1 phase to S phase [34]. The binding of rapamycin with FKBP (FK506 binding protein) will inhibit the activity of mTOR and block PI3K/AKT/mTOR signaling pathway, thereby preventing phosphorylation of S6 K1 and 4E–BP1. As a result, translation of a portion of intracellular proteins is depressed, accompanied by a disturbed cycline protein expression as well as the inhibition on the activity of cyclin-dependent kinase (CDK), which will eventually lead to the block of late G1 phase-S phase transition. In this study, TUNEL staining showed an evidently suppressed apoptosis of granulosa cells in mature antral follicles in CY + Rapa group as compared to CY alone group, along with the absence of apoptotic death of primordial follicles, indicating that rapamycin can inhibit CY-induced apoptosis of follicular granulosa cells in ovaries of mice.

AMH, a glycoprotein secreted by granulosa cells of preantral follicles and small antral follicles, can prevent premature follicle depletion and protect ovarian reserve by regulating oocyte maturation and inhibiting follicular development. The level of serum AMH can be used to evaluate the number of follicles in growing stage and, as an indirect parameter, to estimate the number of primordial follicles. AMH is expected to be employed as a new index for assessment of ovarian toxicity of chemotherapeutics, as a sharp decline even larger than that of estradiol and inhibin B which are usually observed during chemotherapy [35]. Besides, the high sensitivity and specificity as well as the satisfying intraperiodic and interperiodic stability make AMH the only ovarian reserve marker that can be determined in both of follicular phase and luteal phase. Serum AMH determination using ELISA in the present study revealed insignificant inter-group AMH changes among different CY dose groups, in contrast to a dramatic drop in serum AMH level after 4-week CY treatment (*P* < 0.05), suggesting that chemotherapeutics generally show an impact on ovarian reserve only at a certain level of accumulated dose. An insignificant decrease without statistical significance (*P* > 0.05) in serum AMH was observed after concomitant use of cyclophosphamide and rapamycin, which may be associated with the sensitivity and specificity of ELISA as well as some other factors. Therefore, more parameters should be included and verified to evaluate the post-chemotherapeutic protective effect of rapamycin on ovarian reserve.

In this study, determination of the proteins involved in signaling pathways was also performed in an attempt to further explore the potential mechanism of rapamycin on ovarian reserve protection, with the results showing no significant inter-group difference in p-Akt/Akt ratio and p-mTOR/mTOR ratio but an evidently decreased p-rpS6 phosphorylation in CY + Rapa groups as compared to CY groups (*P = 0.001*). These results imply mTOR as the action site through which rapamycin can inhibit its activity for phosphorylation, block the mTOR signaling pathway, prevent the phosphorylation of S6 K and other relevant downstream proteins, and restrain the excessive cell growth and proliferation. Also, these findings are considered as an indirect prove for the hypothesis that cyclophosphamide may lead to excessive activation of primordial follicles through over-activating this signaling pathway.

In addition, we conducted a control experiment using in-vitro cultured ovarian tissues from neonatal mice to investigate the direct impact of rapamycin on the recruitment of primordial follicles. After 1 week of in vitro culture in our preliminary test using 3-day old mice, recruitment of a small number of primordial follicles into primary follicular phase was observed in the ovarian tissues cultured under a natural in vitro condition, which was a striking contrast to the resting state observed in all primordial follicles cultured in the media with rapamycin added. These findings provide a further support for the hypothesis that rapamycin can protect ovarian reserve by inhibiting the activation of primordial follicles.

Studies have reported that single use of rapamycin can impose an anti-tumor effect, whereas combined chemotherapy can further increase the sensitivity of tumor cells to chemotherapy [36], and thus always demonstrates a better antitumor and chemosensitization effect. However, considering the complexity and variability of intracellular signaling pathways, the interaction between rapamycin and antineoplastic agents, as well as the side effects of rapamycin, more fundamental and clinical research should be carried out to verify the in vivo effect of rapamycin.

The unremitting exploration on rapamycin used for tumor treatment and follicle growth regulation is likely to unfold a new path for antitumor treatment and control of chemotherapy-induced POF. Indepth and comprehensive investigations in this field may create a promising future for females undergoing chemotherapy due to the possibility that the fertility of the patients may be preserved without compromising the treatment effect.

## Conclusion

Our study reveals that CY increased phosphorylation of key proteins that play a critical role in regulating the proliferation of oocytes and granulosa cells in primodial follicles through the Pl3K signaling pathway. The specific mTORC1 inhibitor rapamycin can maintain the follicle reserve by preventing the over-activation of primordial follicle via Pl3K/Akt/mTOR signaling pathway. Our findings have revealed, for the first time, an effective protection of ovarian function during chemotherapy by rapamycin, and provide a new nonsurgical method to preserve primordial follicles for female cancer patients. These results may also have implications in ovarian reserve protection and POF prevention.

## Abbreviations

AMH: Serum anti-Müllerian hormone
CMC: Carboxymethyl cellulose
CY: Cyclophosphamide
DMSO: Dimethyl sulfoxide
ELISA: Enzyme-linked immunosorbent assay
GnRHa: Gonadotropin releasing hormone analogue
HE: Hematoxylin-eosin
IACUC: Institutional Animal Care and Use Committee
mTOR: Mammalian target of rapamycin
mTORC1: Mammalian target of rapamycin complex 1
PBS: Phosphate buffered saline
PFA: Paraformaldehyde
PI3K: Phosphatidylinositol 3-kinase
POF: Premature ovarian failure
POI: Premature ovarian insufficiency
Pten: Phosphatase and tensin homolog deleted on chromosome ten
Rapa: Rapamycin
rpS6: Ribosomal protein S6
Tsc1: Tuberous sclerosis complex 1
Tsc2: Tuberous sclerosis complex 2
TUNEL: TdT-mediated dUTP nick end label
